# Supplementary material for: Comparison of the Cisterna Maturation-Progression Model with the Kiss-and-Run Model of Intra-Golgi Transport: Role of Cisternal Pores and Cargo Domains
Source: Int J Mol Sci. 2022 Mar 25;23(7):3590. doi: 10.3390/ijms23073590 (PMC8999060; doi:10.3390/ijms23073590)
Supplement: Supplementary file 1 [file ijms-23-03590-s001.zip › supplementary information.pdf]

Supplementary Information

Video 1. Cartoon showing schematically the mechanisms of intra-Golgi transport according to the cisterna maturation-progression model. Initially, two ER-Golgi carriers are shown in the upper part of the picture. These carriers contain a mega- cargo (blue lines) and a soluble secretory cargo (red dost). The carriers arrive at the cis-side of the Golgi, fuse and form a new cis-cisterna containing mega-cargo and soluble cargo. Coatomer I (black dots) forms COPI-coat. COPI-coat is formed and COPI-coated buds on cisternal rims of all Golgi cisternae appear. These buds become enriched in corresponding to their cisterna Golgi enzymes. Then COPI-coated buds become vesicles. During their fission these buds are transformed into vesicles enriched in Golgi enzymes and then undergo uncoating. Next, fusion of COPI- dependent vesicles with the proximal cisternae and recycling of Golgi enzymes to the corresponding proximal cisternae are shown. Then there is attachment of coatomer I to cisterna rims. The consecutive event is the arrival of new ER-Golgi carriers and formation of a new cis–cisterna, the vesicle fission and formation of the empty trans- cisterna. (I) Fusion of the carriers with the formation of the new cis-cisterna, fusion of COPI-dependent vesicles with the proximal cisternae and formation of two post-Golgi carriers from the empty trans-cisterna are shown. Then consecutive rounds of the same process within the distal part of the Golgi are presented.

Video 2 represents the schematic cartoon explaining the asymmetric KARM, which poses that the distal compartment 2 is composed of the main part and smaller part where the cargo is concentrated. These two parts are connected with a thin tubules which could be easily broken when lipids from the compartment 1 would diffuse to it. Thus, KARM suggests that compartment should be initially fused and then due to some reasons, the tubule would undergo fission. Additional demand for asymmetrical KARM is the necessity of the mechanisms responsible for the change of the cargo in such a way, which would induce the higher ability of our cargo to form temporal aggregates at the distal compartment. These temporally existing aggregates would be not able to diffuse through the thin tubule backword. There are several events, namely, formation of the SNARE complex composed of V and T-SNAREs (brown and magenta lines to the left) between the mega-cargo (blue lines) containing cisterna distension and the rim of the distal cisterna; fusion between the distension and the rim; integration of the distension into the distal cisterna; elongation of the first cisternae; replacement of the SNAREs and formation of the new SNARE complex composed of another set of SNAREs (red and green line); fusion of the cargo containing distension situated within the second cisterna with the rim of the third cisternae containing a pore; additional rounds of fusion/fission processes. Finally, a mega-cargo-containing distension (white arrow) localized in the first medial cisternae and separated from the rest of this cisternae with row of pores is also connected (red arrow) with the cis-most cisterna (yellow).
